# Huge parathyroid carcinoma: Clinical considerations and literature review

**DOI:** 10.1186/1477-7819-3-39

**Published:** 2005-06-23

**Authors:** Maria Grazia Chiofalo, Francesco Scognamiglio, Simona Losito, Secondo Lastoria, Ugo Marone, Luciano Pezzullo

**Affiliations:** 1 Department of Surgical Oncology, National Cancer Institute of Naples, Naples, Italy; 2 Unit of Thyroid and Parathyroid Surgery, National Cancer Institute of Naples, Naples, Italy; 3 Department of Pathology, National Cancer Institute of Naples, Naples, Italy; 4 Department of Nuclear Medicine, National Cancer Institute of Naples, Naples, Italy

## Abstract

**Background:**

Parathyroid carcinoma is a rare malignancy, with an incidence of 0.5 to 4% of all cases of primary hyperparathyroidism. Surgery is the only curative treatment.

**Case presentation:**

We report the case of a 66-year-old man referred for a large suspicious substernal goitre associated with severe hypercalcemia due to hyperparathyroidism. After normalization of serum calcium levels, patient underwent surgery. The voluminous cervicomediastinal firm mass could not be removed through the cervical incision; therefore a cervicothoracic approach was employed. Histopathology revealed a giant parathyroid cancer of 450 grams. A review of the literature was also undertaken to summarize the current treatment approaches for this rare malignancy.

**Conclusion:**

Parathyroid cancer is usually not recognized either preoperatively or intra-operatively. *En bloc *resection of the tumour with the adjacent tissue is the treatment of choice and it is very important to avoid the rupture of the capsule during operation. Neither tumour size, nor the lymph node status appears to play a role in prognosis. The management of parathyroid carcinoma is a challenge even for experienced surgeons.

## Background

Parathyroid Carcinoma (PC) is a rare malignancy with an incidence of 0.5 to 4% in all patients surgically treated for primary hyperparathyroidism [1,2]. Generally the tumour is solitary, although recently a double parathyroid carcinoma has been reported [3]. This tumour has a high probability to recur locally and to spread to regional nodes and distant sites. It is difficult to diagnose parathyroid cancer preoperatively. Often local invasion at operation and subsequent histological examination reveal malignancy and differentiation from a benign parathyroid adenoma can be difficult even after histological examination. However, the presence of some signs such as profound hypercalcemia, markedly elevated levels of parathormone and a clinically palpable cervical mass can raise suspicion preoperatively of a malignant lesion [1,2,4,5].

We report the case of a patient admitted to our department for a large substernal goitre suspected to be a thyroid cancer, associated with severe hyperparathyroidism.

## Case presentation

A 66-year-old man was referred with a diagnosis of hyperparathyroidism, mild renal failure, peptic ulcer and large suspicious cervico-mediastinal goitre. The patient had fatigue, anaemia, anorexia and bone pain for several months. The fine needle aspiration of the neck mass, performed before the admission showed the presence of malignant cells. Preoperative laboratory findings are reported in table 1.

**Table 1:** Laboratory values

	**Preoperative laboratory values**	**Postoperative laboratory values**
Creatinine	2.30 mg/dl (0.60 – 1.30)	4.00 mg/dl (0.60 – 1.30)
ALP	825 U/L (50 – 140)	500 U/L (50 – 140)
Calcemia	14.2 mg/dl (8.4 – 10.5)	6.3 mg/dl (8.4 – 10.5)
Phosphorous	3.1 mg/dl (2.5 – 5.0)	2.4 mg/dl (2.5 – 5.0)
PTH	1828 pg/ml (10.0 – 65.0)	20.9 pg/ml (10.0 – 65.0)

ALP alcaline phosphatase PTH parathyroid hormone

**Figure 1:**
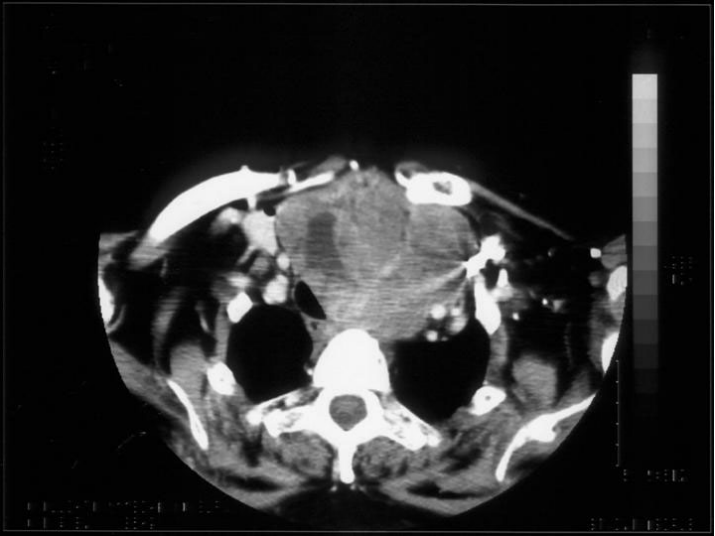
CT Scan showing a large solid left neck mass, with extension to the mediastinum and displacement of the trachea, oesophagus and great vessels.

Ultrasonography of the neck showed an enlarged thyroid gland with substernal extension; many solid nodules and some calcifications were present. The computerised tomographic (CT) scan showed a large solid mass in left neck, with extension to the mediastinum, and displacement of the trachea, oesophagus and great vessels (Figure 1). Tracheobronchoscopy demonstrated substenosis of the proximal trachea due to extrinsic compression.

**Figure 2:**
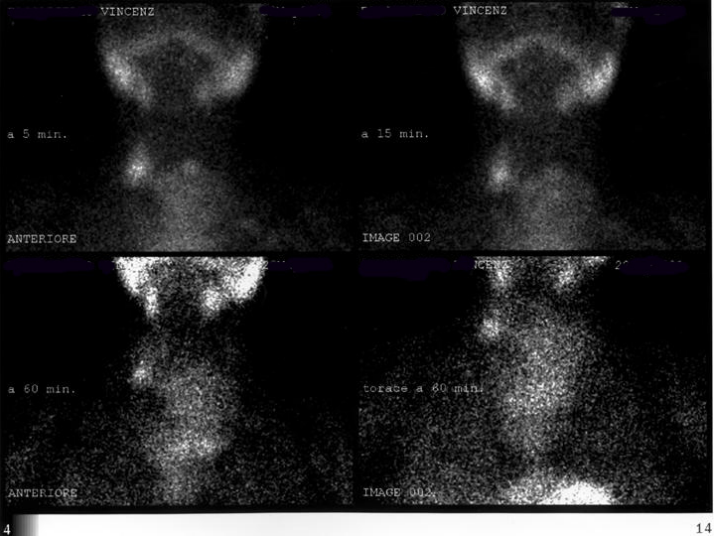
Sestamibi scintigraphy: diffuse enlargement of the thyroid gland associated with irregular uptake of the radionuclide.

Tc-99m sestamibi scintigraphy demonstrated diffuse radiopharmaceutical uptake within the thyroid region and the lower portion of the neck. This tracer accumulation corresponded to the mass and it was persistent during the different phases of the scintigraphy without significant changes (Figure 2). The lack of a spot area of increased MIBI uptake ruled out the diagnosis of adenoma. The scintigraphic pattern was classified as due to a multinodular goitre.

The patient was vigorously hydrated and bisphosphonates were given preoperatively to control hypercalcemia. Eight days later the serum calcium level decreased to 10.4 mg/dl. With the above clinical findings, the patient underwent surgical neck exploration.

At surgery a voluminous cervicomediastinal firm mass was found that could not be removed via the superior thoracic strait; therefore a complete sternotomy was performed. The surgical findings revealed an ovoid, grayish-yellow, hard mass, measuring 12 × 9 × 8 cm, adherent to the base of the left lobe of the thyroid, extending to the anterior upper mediastinum; the recurrent laryngeal nerve was laterally displaced but not invaded. Therefore *en bloc *resection of the mass along with the total thyroid gland and the connective tissue apparently involved in the tumour was performed. All the lymphoid tissue from the ipsilateral tracheoesophageal groove to the upper mediastinum was removed

**Figure 3:**
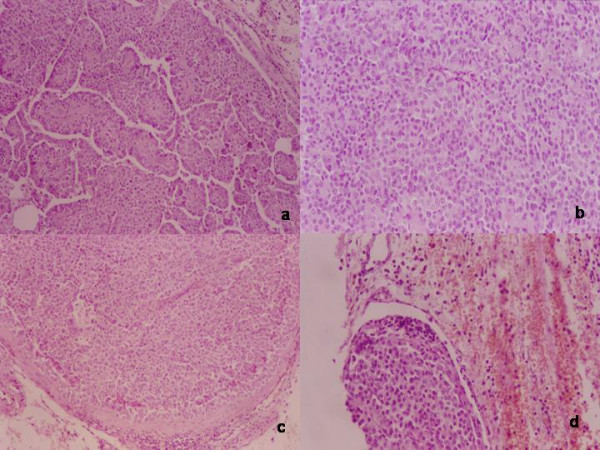
Histological findings: H/E a) papillary growth pattern, focal nuclear pleomorphism and multinucleate giant cells; b) diffuse, sheet like, growth pattern c) massive infiltration in an encapsulated nodule, suggesting metastatic lymphnode; d) vascular invasion in the soft tissue surrounding the parathyroid;

The histological report described a parenchymatous thyroid gland, with a subcapsular gelatinous nodule (1 cm diameter). The voluminous tumour adherent to the base of left lobe was multinodular, greyish-yellow. Histologically many fibrous bands of acellular collagenous tissue extending from the thickened capsule, subdivided the neoplasm into irregular compartments. The tumour cells, arranged in trabecular and solid sheets, were homogeneous; they showed round – ovoid nuclei, with evident nucleoli, and clear cytoplasm; occasionally cytoplasm was abundant, eosinophilic and granular; nuclei were pleomorphic; mitoses were present (3 per 10 HPF). Rare multinucleated osteoclastic-like giant cells were found around foci of haemorrhage; necrosis and coarse calcifications were observed. Both capsular and vascular invasion were present at the periphery of neoplasm. In the infiltrated adjacent adipose tissue, well-circumscribed, encapsulated nodules of tumour, with a peripheral rim of lymphoid tissue were found; they were considered to be metastatic lymph nodes. Final diagnosis was a parathyroid carcinoma (Figure 3a, 3b, 3c, 3d). The postoperative course was complicated by transient oligouria, with worsening the chronic renal failure.

**Figure 4:**
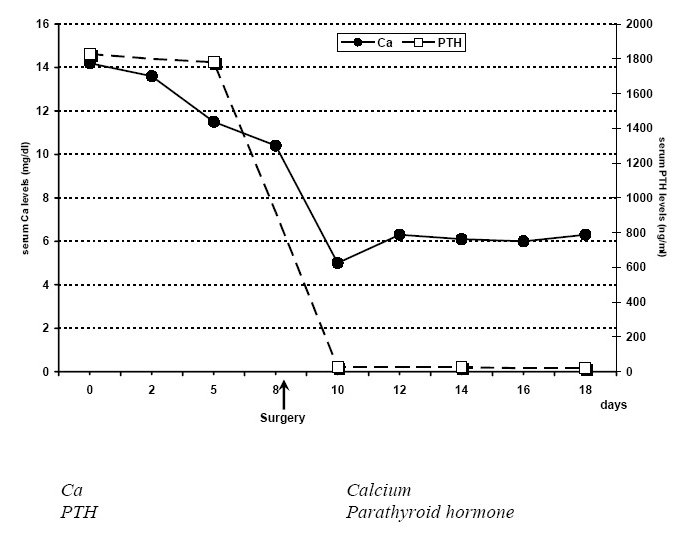
Course of serum Ca and PTH levels during patient's hospitalization.

The patient also had severe postoperative hypocalcaemia, necessitating calcium and vitamin D therapy. The postoperative laboratory values at hospital discharge are shown in table 1. Figure 4 shows the change of serum Calcium and PTH levels during patient's hospitalization

At follow-up, three, six, nine and 12 months after surgery, the patient was disease-free with normal levels of PTH and calcaemia and improvement of the bone disease symptoms. Whole body positron emission tomography (PET) performed one year after surgery was negative.

## Discussion

Parathyroid carcinoma is a rare disease and the preoperative diagnosis is often difficult because clinical manifestations are similar, although more often severe than in patients with parathyroid adenomas. Parathyroid carcinomas are generally larger and firmer than parathyroid adenomas. The clinical manifestations of hyperparathyroidism are usually more severe, and serum levels of calcium, PTH and alkaline phosphatase are significantly higher than in patients with adenoma. Patients with parathyroid carcinoma have a high incidence of renal dysfunction, osteoporosis and gastrointestinal symptoms [1,2,4].

Preoperative and intraoperative suspicion is important in planning the effective surgical treatment: the complete resection of the tumour at the first neck exploration is reported to provide the best chance of cure. The optimal surgical treatment is *en bloc *resection with ipsilateral thyroid lobectomy and removal of any enlarged or abnormal lymph nodes. Most of the authors emphasize the importance of an aggressive initial approach in reducing local and distant recurrences [4,6-12]. Particular attention must be given to avoid rupture of the tumour during surgery, because of the very high risk of local seeding and persistent or recurrent disease [4,13]; in these cases, local surgical excision is useful for palliation but is rarely curative [14,15].

The management of the recurrent laryngeal nerve is more controversial but it should be resected only when it is not functioning preoperatively or is invaded by tumour [1,4,15,16]. The clinical behaviour of parathyroid carcinoma is variable, some patients are cured, others have an indolent course; recurrent or persistent disease occurs in about 50% of cases; usually the tumour recurs within 3 years after initial operation, although much longer intervals have been described [4,6,17,18]. Surgery is the treatment of choice even for recurrent and persistent disease when the site of tumour can be localized: lung metastases are the most frequent type of recurrences after local recurrences; Hundley *et al*, have recently reported a case of resection of multiple PC lung metastases 20 years after the initial neck surgery, with a significant improvement of clinical and metabolic complications after removal of metastatic parathyroid cancer from both lungs [19].

Surgery is also important to palliate hypercalcemia-associated metabolic complications and symptoms and to prolong survival [4,6,7,10,15,20,21].

Recently some authors have reported a role for adjuvant radiation therapy. Neck and mediastinum adjuvant irradiation has been reported to be useful in reducing the risk of loco-regional disease progression and in improving survival [16,22-24]. Other reports suggest a positive effect of radiotherapy for palliating symptoms of hypercalcaemia in inoperable patients [23-25].

In our case, although the laboratory data revealed marked hyperparathyroidism and the patient had all the signs and symptoms of severe hyperparathyroidism, none of the preoperative localizing studies raised suspicion of the presence of a parathyroid carcinoma. The slow growth of the mass and the preoperative FNAB suggested a large substernal goitre with malignant transformation; only the postoperative histological exam revealed a diagnosis of parathyroid cancer.

This is an interesting case because of the large size of the parathyroid cancer with regional nodal metastases without any evidence of distant metastases or local recurrence one year after initial surgery. In the literature parathyroid cancers ranges from 1.5 to 6 cm in diameter and from 1.5 to 27 grams in weight [18,26]. A case of giant paratyroid carcinoma of 1200 grams with nodal involvement, presenting as a substernal goitre has been reported; it was "*en bloc*" resected and the patient was alive and disease free after 4 years [27]. The tumour size does not appear to play a role in the prognosis; the larger tumours in fact don't seem more likely to recur than the smaller ones. In the experience recently reported by Clayman neither local invasiveness seems to correlate with likelihood of recurrence [16,28]. Our case too confirms that many biologic factors involved in the clinical course of parathyroid cancer remain unclear. Most of the parathyroid cancers are diagnosed after surgery and often only after reoperation for local or distant recurrence; many patients have a good clinical course despite the type of the initial operation; others have recurrent or persistent hypercalcemia and severe metabolic complications that can be difficult to control and often result in death [15]. The outcome of the patients with parathyroid carcinoma depends on the biologic features and on the management of the recurrent disease more than on initial therapy. In the future an understanding of molecular pathogenesis of this rare malignancy could provide a means for early diagnosis and new treatment strategies. [16,29].

## Conclusion

Based on current knowledge, clinicians should consider parathyroid carcinoma in differential diagnosis when patients present with severe hyperparathyroidism and a palpable cervical mass. When there is an intraoperative suspicion of malignancy, a complete surgical resection should be undertaken with special attention to avoid the rupture of the capsule of the tumour. We believe this approach offers the best chance for a successful outcome.

## Competing interests

The author(s) declare that they have no competing interests.

## Authors' contributions

MGC Conceived the study, carried out the literature search, and draft the manuscript

FS SL UM helped in management of the patient and preparation of the manuscript

SL Carried out the preoperative scintigraphy and helped in preparation of the manuscript

LP Shaped the idea for the manuscript, coordinated the study and edited the manuscript

All authors read and approved the final manuscript.
